# Use of Thermodynamic Orientors to Assess the Efficiency of Ecosystems: A Case Study in the Lagoon of Venice

**DOI:** 10.1100/tsw.2002.88

**Published:** 2002-01-29

**Authors:** Simone Bastianoni

**Affiliations:** Department of Chemical and Biosystems Sciences, University of Siena, Via A. Moro, 53100 Siena, Italy

**Keywords:** orientors, exergy, emergy, exergy/emergy ratio, lagoon of Venice

## Abstract

So-called orientors have been introduced at the interface between ecology and thermodynamics. Two have been chosen here to compare the characteristics of five ecological systems: exergy, which is related to the degree of organization of a system and represents the biogeochemical energy of a system, and emergy, which is defined as the total amount of solar energy directly or indirectly required to generate a product or a service. They represent two complementary aspects of a system: the actual state and the past work needed to reach that state. The ratio of exergy to the emergy flow indicates the efficiency of an ecosystem in producing or maintaining its organization.

The main system under study is a portion of the Venice Lagoon, which is used as a fish farming basin. Four other aquatic ecosystems were considered for comparison. Results show that the ecosystem within the Venice Lagoon is the one with the highest efficiency in transforming the available inputs in organization of the system. This fact is due to human intervention, which is very limited but also very effective.

## INTRODUCTION

The system analyzed in this study is a fish farming basin in the central part of the lagoon of Venice; in the same part of the lagoon, there are other such basins that occupy a total of 9000 ha. Fish farming basins consist of peripheral areas of lagoon surrounded by banks in which local species of fish and crustaceans are raised. Salt water from the sea and freshwater from canals and rivers are regulated by locks and drains. Control of water levels, salt content, and drainage toward the sea are part of an ancient tradition, which is an economic and cultural heritage.

People learned how to exploit the instinct of certain species of fish that enter the lagoons, delta, and coastal ponds and are attracted by available food and calm waters. In spring, young specimens and adult fish come from the sea; traditionally, they were herded into the basin, where they grew quickly in the shallow nutrient-rich waters. The same principles are exploited today; however, the basins are stocked with artificially raised juveniles or fry. In autumn, the fish would normally return to the sea, attracted by the warmer water and to reproduce. Instead, they are directed into special structures that act like traps by regulating the flow of water, where they are selected on the basis of size and type. The fish of highest demand raised in basins are Dicentrarchus labrax (bass) and Sparus auratus. Various types of mullet are also raised, as well as eels and mollusks.

Environmental monitoring provided data on the biotic (macrophytes, macrozoobenthos, phytoplankton, and zooplankton) and abiotic (sediment and water column) components. Fish production was also monitored where changes had been noticed in the last 10 years, namely, an increase in the fraction of *S. auratus* and a decrease in mullet and eels, due to deterioration of water quality in the open lagoon.

The ecosystem of Figheri basin was analyzed using thermodynamic functions for open systems. These orientors are "ecosystem properties that generally are optimized during the development of ecosystems. They are consequences of self-organizing processes and as such they are able to indicate the integrity of the system … [and] are capable of being used to describe ecosystem states as holistic, functional units"[1].

Specifically, we used *emergy*, which expresses the memory of the flows that went into creating the present state of the system, and *exergy*, which is a measure of the present state, organization, and information content of the system, or how far the system is from thermodynamic equilibrium.

## METHODOLOGY

Emergy has been defined as "the available solar energy previously used up directly and indirectly to make a service or a product"[2,3]. Its units are solar energy joules (seJ). The "intensive" numeraire related with emergy is the *transformity* defined as the solar energy used up (directly and indirectly) to obtain a unit of a product. Emergy analysis is used to establish a longer-term sustainability and a measure of environmental stress. It considers a system with larger boundaries, including all the inputs that contributed to form a product, including environmental inputs that are regarded as free in energy analysis. Emergy accounts for nature's "labor" necessary to obtain a given product or flow. If an input has a high emergy content, it is likely to be a key factor in the production process. Since much "labor" is required for its renewal (it needs a large amount of space and/or time and/or energy), it may become a limiting factor.

Exergy is the maximum work that can be obtained from a system when the system is brought from its present state to the state of thermal, mechanical, and chemical equilibrium with the surrounding environment. Exergy can be written as the weighted sum of three gradients (of intensive variables), where the weight is the corresponding extensive variable:

$$dEx = S\,dT - V\,dp + \sum_i N_i\,d\mu_i$$

where S is entropy, V is volume, and $N_i$ is the number of molecules of the $i^{th}$ species (extensive variables) and dT, dP, and $d\mu_i$ are gradients of intensive variables (temperature, pressure, and chemical potential, respectively, of the $i^{th}$ species) between the system and the environment.

Since living systems have an information content that is vastly different from the mere concentrations of chemical substances, it is necessary to adapt the definition of exergy to their basic characteristics. Jørgensen proposed classifying ecosystem components according to their complexity on the basis of the number of genes in the DNA of the species that compose them. By applying this approach to an ecosystem (for example, an aquatic environment containing phytoplankton, P, zooplankton, Z, fish, F, and detritus, D), the following formula can be obtained[4]:

$$Ex = RT\ [P(1.79\cdot10^{6}) + Z(1.05\cdot10^{8}) + F(2.52\cdot10^{8}) + (D+P+Z+F)\ 7.34\cdot10^{5}] \quad [J/l]$$

The approximations used enable this formula to be utilized to obtain a relative, but not absolute value of exergy. In other words, the values can be used to compare ecosystems or the same ecosystem at different times.

Emergy and exergy are complementary concepts, the former based on the history of the system and the latter examining the actual state. When a system follows a process of selection and organization, one can use the ratio of exergy to emergy flow in order to assess the efficiency of an ecosystem in transforming available inputs in actual information and organization[5]. The higher the ratio, the greater the efficiency of the ecosystem in transforming available inputs (as emergy) into structure and ecosystem organization (as exergy). Its units are $J\cdot year\cdot seJ^{-1}$. Since dimensions are those of time, they cannot be regarded as real efficiency (which is dimensionless), but more as an index of efficiency[6].

## RESULTS AND DISCUSSION

### Emergy Evaluation

The flows of local renewable resources (solar energy, rain, wind, tides, and geothermal heat) were calculated for the total area of the basin. Solar energy was calculated from the insolation of the area minus 20% for albedo. Rain was calculated from mean rainfall data. Assuming that the same volume of water enters and leaves the lagoon in a year, freshwater entering through the Lova and Novissimo canals was estimated as the difference between evaporation and rainfall, because the average water level is constant.

Each year, the basin is drained to remineralize the nutrients and is flushed several times with water from the lagoon. The quantity of water entering was calculated from the rise in water level during flushing. The maintenance costs of the basin, including fuel, electricity, and labor, were transformed into emergy. The total emergy flow, obtained by summing all the independent contributions, made it possible to calculate the transformity of fry and fish, given the quantities produced.

Table 1 shows the emergy evaluation. Emergy is given as the total of renewable, local nonrenewable, and imported nonrenewable inputs. Renewable input is calculated as the sum of rain, tides, geothermal heat, and freshwater from the lagoon. This research considers only the largest (rain) of the three (sun, rain, and wind) inputs of Figheri basin (see Table 1), because the inputs are all coproducts of the same process, namely, the flow of solar energy to the Earth. This avoids counting the same phenomenon twice under different names[2]. Freshwater is regarded as a totally renewable input, since at the end of the cycle, it goes into the lagoon. It can be regarded as a temporary storage of water from the canal and is therefore renewable.

### Exergy Evaluation

Only the interior of the Figheri basin was analyzed, because it was difficult to obtain data, especially for fish, outside the basin. When the Jørgensen equation was applied to Figheri basin, the contribution due to cormorants was also considered, since these predators are increasing rapidly and have a large impact on the ecosystem. Each term was obviously calculated on the basis of estimates and of the many samples (mainly phytoplankton and zooplankton) that were taken.

**TABLE 1**
**Emergy Evaluation of the Figheri Basin**

| Item | Quantity | Units | Solar Transformity (seJ/unit) | Solar Emergy ($10^{16}$ seJ/year) |
|---|---|---|---|---|
| *NURSERY* | | | | |
| Phytoplankton | $3.90 \cdot 10^{6}$ | IT£/year | $1.11 \cdot 10^{9}$ | 0.43 |
| Zooplankton | $1.25 \cdot 10^{7}$ | IT£/year | $1.11 \cdot 10^{9}$ | 1.39 |
| Yeast | $1.60 \cdot 10^{6}$ | IT£/year | $1.11 \cdot 10^{9}$ | 0.18 |
| Food | $3.00 \cdot 10^{6}$ | IT£/year | $1.11 \cdot 10^{9}$ | 0.33 |
| Sea water | $3.23 \cdot 10^{10}$ | g/year | $8.99 \cdot 10^{4}$ | 0.29 |
| Fuels | $1.47 \cdot 10^{12}$ | J/year | $6.60 \cdot 10^{4}$ | 9.67 |
| Electricity | $1.11 \cdot 10^{11}$ | J/year | $2.00 \cdot 10^{5}$ | 2.21 |
| Human labor | $3.01 \cdot 10^{9}$ | J/year | $7.38 \cdot 10^{6}$ | 2.22 |
| *FIGHERI BASIN* | | | | |
| Sunlight | $1.56 \cdot 10^{16}$ | J/year | 1 | 1.56 |
| Rain | $3.16 \cdot 10^{12}$ | g/year | $8.99 \cdot 10^{4}$ | 28.39 |
| Wind | $3.70 \cdot 10^{13}$ | J/year | $1.50 \cdot 10^{3}$ | 5.56 |
| Tides | $6.81 \cdot 10^{12}$ | J/year | $3.02 \cdot 10^{4}$ | 20.57 |
| Earth cycle | $1.32 \cdot 10^{13}$ | J/year | $3.44 \cdot 10^{4}$ | 45.51 |
| Fresh water | $2.61 \cdot 10^{12}$ | g/year | $2.03 \cdot 10^{5}$ | 53.06 |
| Lagoon water | $1.69 \cdot 10^{12}$ | g/year | $8.99 \cdot 10^{4}$ | 15.21 |
| Fuels | $1.27 \cdot 10^{12}$ | J/year | $6.60 \cdot 10^{4}$ | 8.41 |
| Electricity | $8.48 \cdot 10^{11}$ | J/year | $2.00 \cdot 10^{5}$ | 16.96 |
| Human labor | $7.03 \cdot 10^{9}$ | J/year | $7.38 \cdot 10^{6}$ | 5.19 |
| Services | $8.00 \cdot 10^{7}$ | IT£/year | $1.11 \cdot 10^{9}$ | 8.88 |
| *Total emergy* | | | | **218.91** |
| Product: fish | $2.60 \cdot 10^{7}$ | g | $8.42 \cdot 10^{10}$ | |
| | $8.92 \cdot 10^{13}$ | J | $2.45 \cdot 10^{4}$ | |

The biomass of fish was calculated by cohort analysis, considering the fry introduced and mortality due to natural causes and fishing, since *S. auratus* is generally fished at an age of 2 years and bass at an age of 3 years. The number of cormorants and their biomass were estimated from observations and the quantity of fish they are estimated to eat in 1 year. This gave the number of cormorants supported by the Figheri basin ecosystem. The total exergy concentration (Jørgensen's *modo*) estimated for the Figheri basin was $71.2 \cdot 10^{9}$ J/l.

## Exergy/Emergy Ratio and Comparison with Other Ecosystems

The results for the basin of Figheri can be compared with those of four other aquatic ecosystems. Two of the water bodies used for comparison are in North Carolina, U.S., and are part of a group of similar systems constructed to purify urban waste waters. Of the six ponds that compose the system, three are control ponds that receive a mixture of estuarine waters and purified waters from the local sewage treatment plant and three are waste ponds that receive estuarine waters mixed with more polluted, or nutrient-rich, waste waters. Plants and animals were introduced to the ponds to create new ecosystems by natural selection. The different conditions have produced quite different ecosystems in the two types of pond, with a prevalence of phytoplankton and crustaceans in the waste ponds and a great abundance of aquatic plants in the control ponds.

The third water body used for comparison is the lake of Caprolace in Latium, at the edge of the Circeo National Park. This is an ancient natural formation fed mostly by rainwater plus an

**TABLE 2**
**Exergy, Emergy Density, and Their Ratio for Five Ecosystems**

| | Figheri Basin | Lake Caprolace | Control Pond | Waste Pond | Lake Trasimeno |
|---|---|---|---|---|---|
| Emergy density (seJ year$^{-1}$ l$^{-1}$) | $12.2 \cdot 10^8$ | $0.9 \cdot 10^8$ | $20.1 \cdot 10^8$ | $31.6 \cdot 10^8$ | $0.3 \cdot 10^8$ |
| Exergy density (J l$^{-1}$) | $71.2 \cdot 10^9$ | $4.1 \cdot 10^9$ | $1.6 \cdot 10^9$ | $0.6 \cdot 10^9$ | $1.0 \cdot 10^9$ |
| Exergy/emergy (J year seJ$^{-1}$) | 58.5 | 44.3 | 0.8 | 0.2 | 30.6 |

input rich in nitrogen, phosphorus, and potassium that percolates from nearby agricultural land. Human impact is low. A quantity of fish is taken each year, but is not large enough that the fish population decreases[5].

The fourth ecosystem used for comparison is Lake Trasimeno in Umbria[7].

Table 2 shows emergy and exergy density values and ratio. Densities were used to enable comparison between ecosystems in different areas. Figheri basin is an artificial ecosystem, but has many characteristics typical of natural systems. This depends partly on the long tradition of fish farming basins in the lagoon of Venice, which has "selected" the best management strategies.

We had observed that the natural lake (Caprolace) had a higher exergy/emergy ratio than the control and waste ponds due to a higher exergy density and a lower emergy density. These observations were confirmed by the study of Lake Trasimeno that was carried out by a research group at Perugia University[7]. In natural systems where selection has acted undisturbed for a long time, this ratio is higher and decreases with the introduction of artificial stress factors.

The human contribution at Figheri basin manifests as a higher emergy density (of the same order of magnitude as that of artificial systems) than in natural systems. However, there is a striking difference in exergy density, with values of a higher order of magnitude than in any of the other systems used for comparison: Man and Nature are acting in synergy to enhance the performance of the ecosystem. The fact that Figheri can be regarded as a stable ecosystem makes this result even more interesting and significant.

## CONCLUSIONS

Indications useful for the management of the basin and for understanding the role of structures and ecosystems of this type emerge from the present study. On the whole, Figheri basin production is very sustainable. As an ecosystem, it seems to benefit from human intervention rather than be damaged by it, as is usually the case. Human intervention has greatly promoted ecosystem efficiency. Other conclusions can be drawn from the analysis of the various orientors:

1. Emergy analysis indicates that fish production at Figheri basin has an efficient use of local resources and low external input.
2. Exergy analysis shows that the ecosystem of Figheri basin is more organized than the other ecosystems used for comparison by at least one order of magnitude. The organization density (per unit volume of water) was about 70 times that of Lake Trasimeno.
3. The exergy/emergy ratio shows that the ecosystem at Figheri basin has a higher overall efficiency than the others in transforming input into ecosystem components (e.g., fish and zooplankton). Its efficiency is of the same order of magnitude as natural systems but is higher than that of systems with limited human input, such as Lake Caprolace. Its efficiency is two orders of magnitude greater than artificial ecosystems, by virtue of a higher level of organization and less need for external input.

## ACKNOWLEDGMENTS

This research was carried out with the financial and technical support of the Consorzio Venezia Nuova. Many thanks to Dr. Riccardo Strada for his precious information on the Figheri basin ecosystem.
